# Effect of fasting glucose levels on carotid intima-media thickness in premenopausal versus postmenopausal women

**DOI:** 10.20945/2359-4292-2023-0110

**Published:** 2024-03-15

**Authors:** Ren Xia, Su Fan, Hu Jian, Cao Lei, Mei Wendan, Wang Chenxu, Fang Yicheng, Grace Tavengana, Jiang Mingfei, Wu Huan, Wen Yufeng

**Affiliations:** 1 Wannan Medical College School of Public Health Wuhu Anhui Province China School of Public Health, Wannan Medical College, Wuhu, Anhui Province, China; 2 Wannan Medical College School of Clinical Medicine Wuhu Anhui Province China School of Clinical Medicine, Wannan Medical College, Wuhu, Anhui Province, China; 3 Wannan Medical College School of Laboratory Medicine Wuhu Anhui Province China School of Laboratory Medicine, Wannan Medical College, Wuhu, Anhui Province, China

**Keywords:** Postmenopause, premenopause, carotid intima-media thickness, fasting blood glucose

## Abstract

**Objective:**

To investigate the relationship between fasting blood glucose (FBG) and carotid intima-media thickness (IMT) in premenopausal and postmenopausal women.

**Subjects and methods:**

The study enrolled 2,959 women seen at the Maanshan People's Hospital of Anhui Province from December 2013 to December 2018. Carotid IMT was measured using Doppler ultrasound. Linear regression and R smoothing curves were used to analyze the relationship between blood glucose level and carotid IMT in the premenopausal and postmenopausal groups.

**Results:**

Postmenopausal compared with premenopausal women had higher mean IMT (mIMT; 0.81 ± 0.23 mm *versus* 0.70 ± 0.14 mm, respectively, p < 0.001) and maximum IMT (maxIMT; 0.86 ± 0.35 mm *versus* 0.74 ± 0.16 mm, respectively, p < 0.001) values. On linear regression analysis, mIMT values increased with increasing FBG values when FBG level was ≤ 7 mmol/L, but no significance was found between FBG and maxIMT. After stratification by menopausal status, mIMT and maxIMT increased with increasing FBG when FBG was ≤ 7 mmol/L in the premenopausal group. In the postmenopausal group, mIMT and maxIMT increased with increasing FBG. After adjustment for covariate factors, the relationship between FBG and mIMT remained the same as before the adjustment, but when FBG was ≤ 11 mmol/L, the maxIMT increased with increasing FBG. In the stratification analysis, maxIMT increased with increasing FBG when FBG was ≤ 7 mmol/L in the premenopausal group, while both mIMT and maxIMT increased with increasing FBG when FBG was > 10 mmol/L in the postmenopausal group.

**Conclusion:**

Levels of FBG contributed more to increased IMT in postmenopausal than premenopausal women. The influence of FBG was greater on maxIMT than mIMT. Additionally, FBG was helpful in assessing focal thickening of the carotid intima.

## INTRODUCTION

Early detection of atherosclerosis is currently an important topic in medicine, and the carotid intima-media thickness (IMT) is being increasingly used as a noninvasive marker of atherosclerosis (1–3). Atherosclerosis-related vascular complications are the main cause of reduced life quality and expectancy in individuals with diabetes mellitus (4,5). Fasting blood glucose (FBG) is the most ordered test to diagnose diabetes and is mainly influenced by endogenous insulin secretion capacity (6,7). However, epidemiological studies have shown conflicting associations between FBG and cardiovascular events (8,9). Levitzky and cols. analyzed data from 4,058 Framingham Offspring cohort participants and found that impaired FBG (between 5.50 mmol/L and 6.25 mmol/L) was associated with a higher 4-year risk of coronary heart disease or cardiovascular disease in women but not in men (10). Yeboah and cols., analyzing 6,753 participants from the Multi-Ethnic Study of Atherosclerosis (MESA), found that impaired FBG at baseline was not independently associated with incident cardiovascular events after 7.5 years of follow-up (11). To date, the association between FBG and subclinical carotid atherosclerosis in Asian populations has not been fully studied.

The menopausal transition is a period of rapid change in physiologic characteristics, including endogenous sex steroid hormones, body composition and fat distribution, and lipid and metabolic profiles (12,13). These changes suggest a potential association between blood glucose levels and menopause (14). Studies have shown that menopause, but not age, is an independent risk factor for fasting plasma glucose levels in women without diabetes (15,16). However, the 8-year Australian Longitudinal Study on Women's Health (17) and the 3-year Diabetes Prevention Program (18) found no association between natural postmenopausal status and diabetes risk. This indicates that the relationship between menopause and diabetes remains controversial. Therefore, this study explored the role and significance of FBG levels in premenopausal and postmenopausal women with diabetes mellitus and stroke.

## SUBJECTS AND METHODS

### Study design and population

This cross-sectional study was conducted in Maanshan People's Hospital and included women who underwent carotid artery examination in the institution's Ultrasound Department from December 2013 to December 2018. The study included 2959 women, of whom 855 (21.6%) were premenopausal and 2104 (71.1%) were postmenopausal. The inclusion criteria were (1) women aged 18-80 years, (2) examination of both carotid arteries, and (3) available data on FBG, mean IMT (mIMT), maximum IMT (maxIMT), age, coronary heart disease (CHD), hypertension, dyslipidemia, smoking, alcohol consumption, body mass index (BMI), total cholesterol (TC), triglycerides (TG), high-density lipoprotein cholesterol (HDL-C), low-density lipoprotein cholesterol (LDL-C), and uric acid (UA). The exclusion criteria included (1) heart disease, (2) cancer, (3) renal insufficiency, (4) history of intracerebral hemorrhage or cerebral thrombosis, and (5) history of carotid artery surgery.

The protocol of the study was approved by the Hospital Review Committee (NO. 2014001). The study was conducted according to the ethical guidelines of the 1975 Declaration of Helsinki, and all study participants signed an informed consent form (19).

### Research methods

#### Questionnaire

The study's questionnaire was developed by radiologists and epidemiologists and was reviewed and revised by clinicians and radiologists. All investigators received uniform training prior to the study. The questionnaire collected data on general demographic and behavioral characteristics, as well as past medical history. All surveys were administered through face-to-face interviews with participants or their accompanying family members.

#### Physical examination, including blood pressure measurement and body mass index calculation

The data were obtained by professionally trained surveyors skilled in operating instruments and equipment according to standard measurement methods. For blood pressure measurement, the participants rested in a quiet environment for 15 minutes. The measurements were obtained at the brachial artery of the right arm at the level of the heart, with the participants sitting down with legs positioned vertically and feet flat on the floor. The measurements were performed using a table mercury sphygmomanometer (Fish Jump, Jiangsu, China). Systolic and diastolic blood pressure were indicated by Korotkoff sounds (phase I and V readings, respectively). Blood pressure was measured three times, with the participant resting for 1-2 minutes between measurements, and the average of the measurements was considered. If the results of the three measurements differed substantially, new measurements were performed. The average of the last two measurements was then used for the analysis (20). For BMI calculation, all participants removed shoes and socks, stood upright, looked straight ahead with straightened chest and arms resting comfortably at their sides, heels together, and heels, hips, and shoulder blades aligned on the same plane. Height and weight measurements are accurate to 0.1 cm and 0.1 kg, respectively. The BMI formula used was weight (in kg) divided by squared height (in m^2^).

#### Carotid ultrasound

The participants were placed in a supine position, with a pillow under the neck, and the neck fully exposed for the examination. The participant's head was tilted to one side during examination of the opposite side. First, we explored the carotid artery bilaterally to rule out the possible presence of atherosclerotic plaque. The specialists performing the examination used 5-13 MHz linear array probes (AlOKA-A7 and AlOKA-A10, Tokyo; and Philips iU22, Colombia), starting at the anterior edge of the sternocleidomastoid muscle and visualizing the proximal, mid, and distal ends of the participant's left common carotid artery and right common carotid artery. Multiangle scans of the left internal carotid artery and right internal carotid artery were performed for visualization of carotid plaques. When one side was finished, the other side was examined to ensure complete inspection. Color Doppler flow imaging was used to measure blood flow, and the average value was calculated from three measurements.

#### Definitions

1. Hypertension: use of antihypertensive drugs, or systolic blood pressure of 140 mmHg, or diastolic blood pressure of 90 mmHg (21).
2. Diabetes mellitus: blood glucose level of 11.1 mmol/L at any time, FPG ≥ 7.0 mmol/L, or glucose level of 11.1 mmol/L at 2 hours in the oral glucose tolerance test (OGTT) (22).
3. Smoking: history of continuous or cumulative smoking for ≥ 6 months (23).
4. Alcohol: consumption of ≥ 50 mL of alcoholic drinks daily and at least 5 times a week (24).
5. Carotid artery disease: presence of a plaque, defined as a focal structure invading the lumen of the artery with at least 0.5 mm, or 50% of the peripheral IMT value, or largest thickness > 1.5 mm when measured from the intima-lumen interface to the media-adventitia interface (25).
6. Carotid IMT: distance between the boundary between the lumen of blood vessels and the vascular endothelial layer, which is identified by double hypoechoic lines that do not protrude into the lumen of blood vessels. The IMT of the distal wall of the right common carotid artery was calculated in the longitudinal axis, according to the method described by Touboul and cols. (26). Echo measurements were used to obtain arithmetic mIMT values and were performed in the following three regions:
  - Proximal zone: about 2 cm above the shunt
  - Distal zone: about 1/2 cm above the shunt
  - Middle area

### Statistical analysis

The statistical analysis was performed using SPSS, version 26.0 (IBM Corp., Armonk, NY, USA). Continuous variables are expressed as median (minimum–maximum) for nonnormally distributed data and mean ± standard deviation for normally distributed data. The Mann-Whitney U, Student's *t*, and chi-square tests were used to analyze differences in demographic characteristics, laboratory tests, and hemodynamic characteristics.

The relationships between FBG and mIMT and between FBG and maxIMT were tested using generalized smoothing splines, and the points ("knots") locations were generated automatically in generalized additive models using the R package MGCV, version (Mixed Generalized Additive Models Computation Vehicle; Wood, 2006). The adjusted factors were age, CHD, hypertension, dyslipidemia, smoking, alcohol, BMI, TC, TG, HDL-C, LDL-C, and UA. According to the location of FBG nodes, the relationships between FBG and mIMT and between FBG and maxIMT in different FBG segments were further analyzed using a linear regression model and adjusted for age, CHD, hypertension, dyslipidemia, smoking, alcohol, BMI, TC, TG, HDL-C, LDL-C, and UA. Postmenopausal stratification was then carried out, and the relationships between FBG-mIMT and FBG-maxIMT were analyzed using generalized smoothing splines, while the relationship between FBG-mIMT and FBG-maxIMT in different FBG segments was analyzed using linear regression according to the node positions before and after stratification. The adjusted factors were age, CHD, hypertension, dyslipidemia, smoking, alcohol, BMI, TC, TG, HDL-C, LDL-C, and UA. The p values were two-sided, and the significance level was set at p < 0.05.

## RESULTS

### Basic characteristics of premenopausal and postmenopausal participants

As shown in Table 1, mIMT, maxIMT, FBG, LDL-C, UA, hypertension, diabetes mellitus, and smoking differed significantly (p < 0.05) between the premenopausal and postmenopausal groups.

**Table 1:** Anthropometric, biochemical, and ultrasound findings of the participants in the premenopausal and postmenopausal groups

Findings	Premenopausal group (n = 855)	Postmenopausal group (n = 2104)	Z/t/χ^2^	p
mIMT (mm)	0.70 ± 0.14	0.81 ± 0.23	-15.289	<0.001
maxIMT (mm)	0.74 ± 0.16	0.86 ± 0.35	-13.535	<0.001
FBG (mmol/L)	7.49 ± 3.61	6.61 ± 3.09	6.216	<0.001
TC (mmol/L)	4.56 ± 1.10	4.50 ± 1.18	1.166	0.244
TG (mmol/L)	1.73 ± 1.44	1.66 ± 1.36	1.264	0.207
HDL-C (mmol/L)	1.2 (0.44-3.01)	1.23 (0.52-3.08)	-1.639	0.101
LDL-C (mmol/L)	2.48 (0.65-6.86)	2.42 (0.38-8.21)	-2.376	0.018
UA (μmol/L)	267.15 ± 77.03	295.68 ± 93.75	-8.049	<0.001
BMI (kg/m^2^)	24.03 ± 3.66	23.97 ± 5.90	0.250	0.802
Hypertension (%)	396 (46.32)	1362 (64.73)	85.523	<0.001
Dyslipidemia (%)	235 (27.49)	614 (29.18)	0.856	0.355
Diabetes (%)	412 (48.19)	877 (41.68)	10.462	<0.001
Smoking (%)	49 (5.73)	83 (3.94)	4.551	0.033
Alcohol (%)	67 (7.84)	136 (6.46)	1.792	0.181

Abbreviations: BMI, body mass index; FBG, fasting blood glucose; HDL-C, high-density lipoprotein cholesterol; LDL-C, low-density lipoprotein cholesterol; mIMT: mean intima-media thickness; maxIMT, maximum intima-media thickness; t, Student's *t* test; TC, total cholesterol; TG, triglycerides; UA, uric acid; χ^2^, chi-square test; Z, Mann-Whitney U test.

### Relationship of fasting blood glucose to mean and maximum intima-media thickness

**Figure 1:**
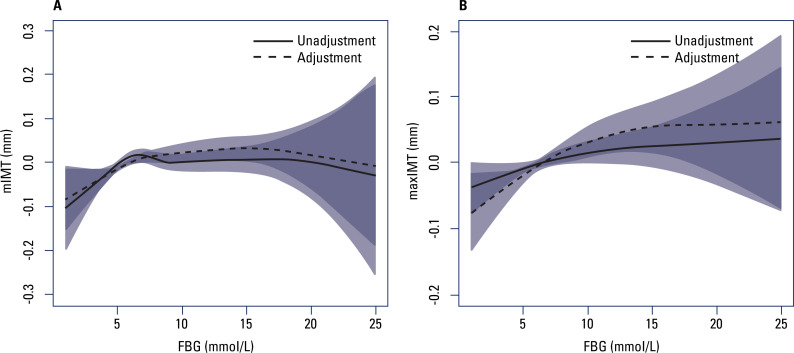
Generalized smoothing spline models of fasting blood glucose (horizontal axis) and **(A)** mean intima-media thickness (vertical axis) and **(B)** maximum intima-media thickness (vertical axis). Solid lines, no adjustment; dotted lines, adjustment for body mass index, total cholesterol, triglycerides, high-density lipoprotein cholesterol, low-density lipoprotein cholesterol, uric acid, hypertension, alcohol, and smoking. The shaded areas indicate the 95% confidence intervals.

Figure 1 shows the relationship of FBG to mIMT and maxIMT. After adjustment for BMI, TC, TG, HDL-C, LDL-C, UA, hypertension, and smoking, mIMT increased with increasing FBG for FBG levels ≤ 7 mmol/L; for FBG levels > 7 mmol/L, no significant relationship was observed between FBG and mIMT. Similarly, maxIMT increased with increasing FBG for FBG levels ≤ 11 mmol/L; for FBG levels > 11 mmol/L, no significant relationship was observed between FBG and maxIMT. The results of the linear regression analysis are shown in Table 2.

**Table 2:** Linear regression analysis of the relationship between fasting blood glucose and mean and maximum intima-media thickness

	FBG (mmol/L)	Unadjusted				Adjusted			
		β	SE	t	p	β	SE	t	p
mIMT (mm)	≤7	0.017	0.004	4.139	<0.001	0.022	0.005	0.005	<0.001
	>7	0.002	0.002	0.973	0.331	0.003	0.002	1.233	0.218
maxIMT (mm)	≤11	0.008	0.003	2.265	0.024	0.015	0.004	3.349	0.001
	>11	-0.002	0.010	-0.234	0.815	-0.003	0.010	-0.251	0.802

Abbreviations: FBG, fasting blood glucose; mIMT, mean intima-media thickness; maxIMT, maximum intima-media thickness. The variables included in the adjusted model were body mass index, total cholesterol, high-density lipoprotein cholesterol, low-density lipoprotein cholesterol, uric acid, triglycerides, hypertension, alcohol, and smoking.

### Relationship of fasting blood glucose to mean and maximum intima-media thickness in premenopausal and postmenopausal participants

**Figure 2:**
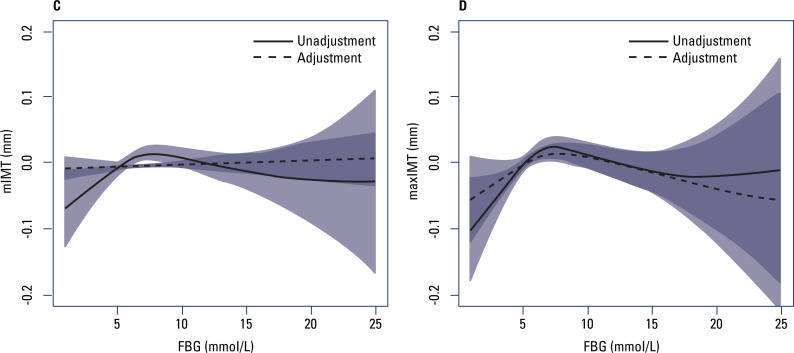
Generalized smoothing spline models of fasting blood glucose (horizontal axis) and (**C**) mean intima-media thickness (vertical axis), and (**D**) maximum intima-media thickness (vertical axis) in the premenopausal group. Solid lines, no adjustment; dotted lines, adjustment for body mass index, total cholesterol, triglycerides, high-density lipoprotein cholesterol, low-density lipoprotein cholesterol, uric acid, hypertension, alcohol, and smoking. The shaded areas indicate the 95% confidence intervals.

**Figure 3:**
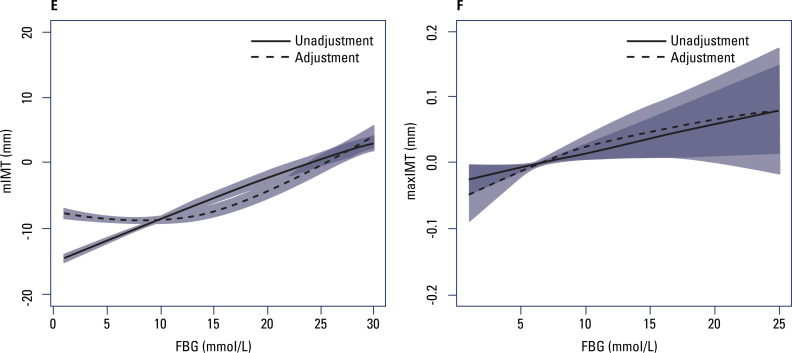
Generalized smoothing spline models of fasting blood glucose (horizontal axis) and (**E**) mean intima-media thickness (vertical axis) and (**F**) maximum intima-media thickness (vertical axis) in the postmenopausal group. Solid lines, no adjustment; dotted lines, adjustment for body mass index, total cholesterol, triglycerides, high-density lipoprotein cholesterol, low-density lipoprotein cholesterol, uric acid, hypertension, alcohol, and smoking. The shaded areas indicate the 95% confidence intervals.

Figures 2 and 3 show the relationship of FBG to mIMT and maxIMT after stratification of the participants into premenopausal and postmenopausal groups with and without adjustment. In the premenopausal group (Figure 2 and Table 3), mIMT increased with increasing FBG for FBG levels ≤ 7 mmol/L before adjustment, but this relationship lost significance after adjustment. In contrast, maxIMT increased with increasing FBG for FBG levels ≤ 7 mmol/L after adjustment; for FBG levels > 7 mmol/L, no significant relationship was observed between FBG and maxIMT. In the postmenopausal group (Figure 3 and Table 4), no significant relationship was observed between FBG and mIMT when FBG levels were ≤ 7 mmol/L, but for FBG levels > 10 mmol/L, mIMT increased with increasing FBG. In contrast, maxIMT increased with increasing levels of FBG.

**Table 3:** Linear regression analysis of the relationship between fasting blood glucose and mean and maximum intima-media thickness in the premenopausal group

	FBG (mmol/L)	Unadjusted				Adjusted			
		β	SE	t	p	β	SE	t	p
mIMT(mm)	≤7	0.033	0.009	3.825	<0.001	0.001	0.001	1.004	0.316
	>7	-0.003	0.002	-1.287	0.199					
maxIMT(mm)	≤7	0.038	0.011	3.637	<0.001	0.041	0.011	3.608	<0.001
	>7	-0.005	0.002	-1.911	0.057	-0.006	0.003	-2.316	0.021

Abbreviations: FBG, fasting blood glucose; mIMT, mean intima-media thickness; maxIMT, maximum intima-media thickness. The variables included in the adjusted model were body mass index, total cholesterol, high-density lipoprotein cholesterol, low-density lipoprotein cholesterol, uric acid, triglycerides, hypertension, alcohol, and smoking.

**Table 4:** Linear regression analysis of the relationship between fasting blood glucose and mean and maximum intima-media thickness in the postmenopausal group

	FBG (mmol/L)	Unadjusted				Adjusted			
		β	SE	t	p	β	SE	t	p
mIMT (mm)	≤10	0.005	0.002	3.329	0.001	0.009	0.004	2.41	0.016
	>10					0.007	0.002	3.389	0.001
maxIMT (mm)		0.008	0.002	3.246	0.001	0.008	0.003	2.959	0.003

Abbreviations: FBG, fasting blood glucose; mIMT, mean intima-media thickness; maxIMT, maximum intima-media thickness. The variables included in the adjusted model were body mass index, total cholesterol, high-density lipoprotein cholesterol, low-density lipoprotein cholesterol, uric acid, triglycerides, hypertension, alcohol, and smoking.

## DISCUSSION

In a previous study, Faeh and cols. demonstrated a close association between blood glucose and carotid IMT by fitting multiple regression models with different sets of risk factors for atherosclerosis and anthropometric variables adjusted for multiple metabolic risk factors (27). Since two values exist for two-sided carotid IMT estimates in practice, it is difficult to determine which IMT measurement should be used. The options for IMT measurements include calculating the average of the maximum values from both sides and different arterial sites; mIMT values over the entire distance are less susceptible to outliers, while maxIMT may reflect a more advanced stage of focal thickening due to plaque formation (26,28,29). To avoid composite scoring, we analyzed the effects of FBG on mIMT and maxIMT separately. Because the incidence of atherosclerosis is much higher in men than women, most studies on the relationship between blood glucose and carotid IMT have focused less on women and more on men and the general population. This study analyzed mainly the relationship between FBG and carotid IMT in premenopausal and postmenopausal women.

Carotid atherosclerosis is an important cause of stroke, and there is growing evidence that hyperglycemia can induce excessive production of mitochondrial reactive oxygen species in cardiovascular cells and that this excessive production can promote atherosclerosis by activating multiple pathways, including increased substrate conversion by aldose reductase, increased formation of methylglyoxal, activation of major advanced glycosylation product precursors, and increased protein modification by O-linked β-N-acetylglucosamine (30,31). The interaction between these factors can increase oxidative stress and proinflammatory reactions, promoting the atherosclerotic process. In addition, hyperglycemia may accelerate atherosclerosis by inducing endothelial cell dysfunction, reducing nitric oxide bioavailability, and promoting vasoconstriction or thrombosis, leading to changes in vascular tissue at the cellular level (32).

In this study, we found that atherosclerosis of the carotid arteries is more susceptible to FBG after menopause. The possible reason for this finding is a marked decrease in estrogen levels after menopause, affecting the protective effect of this hormone on blood vessels. Estrogen exerts bioactive effects mostly through estrogen receptors (ERs), which are widely distributed in vascular smooth muscle and vascular endothelial cells. There are multiple subtypes of ERs; ERα is the main subtype and mediates NO production in estrogen-promoting vascular endothelial cells. Studies have shown that vascular endothelial apoptosis induced by tumor necrosis factor-α (TNF-α) can be reversed by estrogen in a dose-dependent fashion, an effect that can be blocked by ER antagonists (33). In addition, Somjen and cols. found that when endothelial cells from human umbilical veins were treated with estrogen (0.3-300 nm/L), thymine intake increased in a dose-dependent fashion, while ER antagonists could block this effect (34), suggesting that estrogen can promote the synthesis of DNA and vascular endothelial cell growth factor in vascular endothelial cells. Estrogen also activates nitric oxide synthase (eNOS) and promotes NO release through a variety of pathways, including the gene pathway, phosphoinositide 3-kinase-serine/threonine kinase (PI3K-Akt) pathway, and the mitogen-activated protein kinase pathway (35). Estrogen also increases vascular endothelial cell synthesis and prostaglandin I2 (PGI2) release. Additionally, estrogen plays a role in improving vasodilation through regulation of smooth muscle cells, PGI2, NO, and calcium ion expression. Factors that occur in the postmenopausal period, including aging, reduced estrogen levels, vascular endothelial disorders, and NO utilization, among others, may lead to thrombosis. The postmenopausal period is also more likely to be affected by various risk factors and promote the formation of atherosclerosis compared with the premenopausal period. In the present study, mIMT and maxIMT were more likely to be affected by FBG in postmenopausal than premenopausal women. Additionally, the influence of FBG on maxIMT was greater than that on mIMT.

In conclusion, in summary, carotid IMT was more susceptible to FBG in postmenopausal than premenopausal women, and the influence of FBG on maxIMT was greater than that on mIMT. Additionally, FBG was helpful in assessing focal thickening with plaque formation. In women, FBG levels may be a marker of carotid atherosclerotic disease and, eventually, a tool for the prediction and treatment of atherosclerosis.

## Data Availability

the datasets used and analyzed during the current study are available from the corresponding author upon reasonable request.
